# Cytosolic Immunostimulatory DNA Ligands and DNA Damage Activate the Integrated Stress Response, Stress Granule Formation, and Cytokine Production

**DOI:** 10.3390/cells15020139

**Published:** 2026-01-13

**Authors:** Trupti Devale, Lekhana Katuri, Gauri Mishra, Aditya Acharya, Praveen Manivannan, Brian R. Hibbard, Krishnamurthy Malathi

**Affiliations:** 1 Department of Molecular, Cellular and Developmental Biology, University of Toledo, 2801 West Bancroft Street, Toledo, OH 43606, USA; trupti.devale@rockets.utoledo.edu (T.D.); lekhana.katuri@rockets.utoledo.edu (L.K.); gauri.mishra@rockets.utoledo.edu (G.M.); aditya.acharya@rockets.utoledo.edu (A.A.); manivann@umich.edu (P.M.);; 2 College of Medicine and Life Science, University of Toledo, Toledo, OH 43614, USA; 3 Department of Microbiology and Immunology, University of Michigan Medical School, Ann Arbor, MI 48109, USA; 4 School of Medicine, University of Louisville, Louisville, KY 40202, USA

**Keywords:** stress granule, STING, DNA ligands, DNA damage, integrated stress response, cytokine, innate immunity

## Abstract

**Highlights:**

**What are the main findings?**

- The presence of cytosolic dsDNA, exogenous or endogenous, activates the integrated stress response pathway, inducing stress granules and cytokine production.
- The STING-PERK-G3BP1 signaling axis couples DNA sensing with stress and innate immune response pathways.

**What are the implications of the main findings?**

- The cGAS-STING pathway is a valuable target in cancer immunotherapy and pathogen invasion. Combining DNA-damaging agents like radiation or chemotherapy agents with STING activators can enhance cellular response and improve the effectiveness of immunotherapy and host immune response.
- Understanding the modes of STING activation and its role as a central signaling hub that fine-tunes the cell’s response depending on the specific type of cellular threat or stress has implications for developing therapies for diverse pathologies.

**Abstract:**

The presence of aberrant double-stranded DNA (dsDNA) in the cytoplasm of cells is sensed by unique pattern recognition receptors (PRRs) to trigger innate immune response. The cyclic GMP–AMP synthase (cGAS)–stimulator of interferon genes (STING) signaling pathway is activated by the presence of non-self or mislocalized self-dsDNA from nucleus or mitochondria released in response to DNA damage or cellular stress in the cytoplasm. Activation of cGAS leads to the synthesis of the second messenger cyclic GMP–AMP (cGAMP), which binds and activates STING, triggering downstream signaling cascades that result in the production of type I interferons (IFNs) and proinflammatory cytokines. Here, we show that diverse immunostimulatory dsDNA ligands and chemotherapy agents like Doxorubicin and Taxol trigger the integrated stress response (ISR) by activating endoplasmic reticulum (ER) stress kinase, protein kinase RNA-like ER kinase (PERK), in addition to the canonical IFN pathways. PERK-mediated phosphorylation and inactivation of the alpha subunit of eukaryotic translation initiation factor-2 (eIF2α) result in the formation of stress granules (SGs). SG formation by dsDNA was significantly reduced in PERK knockout cells or by inhibiting PERK activity. Transcriptional induction of IFNβ and cytokines, ISR signaling, and SG formation by dsDNA was dampened in cells lacking PERK activity, STING, or key stress-granule nucleating protein, Ras-GAP SH3 domain-binding protein 1 (G3BP1), demonstrating an important role of the signal transduction pathway mediated by STING and SG assembly. Lastly, STING regulates reactive oxygen species (ROS) production in response to DNA damage, highlighting the crosstalk between DNA sensing and oxidative stress pathways. Together, our data identify STING–PERK–G3BP1 signaling axis that couples cytosolic DNA sensing to stress response pathways in maintaining cellular homeostasis.

## 1. Introduction

The innate immune response is the first line of defense, and cells use specialized pattern recognition receptors (PRRs) to recognize unique molecular signatures of pathogens, pathogen-associated molecular patterns (PAMPs), to activate innate immune signaling to produce type I IFN and cytokines [1,2]. The toll-like receptors (TLRs) detect nucleic acids in endosomes, while Rig-I-like receptors (RLRs) and cytosolic DNA receptors (CDRs) recognize viral RNA and DNA genomes and replication intermediates in the cytoplasm, respectively. The cytosolic sensors engage adaptors like mitochondrial antiviral-signaling protein (MAVS) and stimulator of interferon genes (STING) and converge on transcription factors like NF-κB and interferon regulatory factor 3 (IRF3) to transcriptionally induce IFNβ and IFN-stimulated genes (ISGs) [3,4]. Aberrant accumulation of cytosolic DNA, pathogenic or endogenous, is recognized by multiple cytosolic DNA sensors like cyclic GMP-AMP synthase (cGAS), interferon gamma-inducible 16 (IFI16), absent in melanoma 2 (AIM2), DEAD-Box Helicase 41 (DDX41), and Z-DNA-binding protein (ZBP1), which have context-specific preferences based on distribution and features of the ligand [5,6,7,8]. cGAS and cGAS-like receptors are evolutionarily conserved, and in mammals, the cGAS-STING pathway is the major pathway for sensing microbial as well as self-DNA from damaged cells or organelles [9,10,11]. On binding dsDNA, cGAS undergoes a conformational change and dimerization resulting in enzymatic activation, leading to the synthesis of 2′-3′-cyclic GMP-AMP (cGAMP) from GTP and ATP that potently activates the endoplasmic reticulum (ER)-resident PRR adaptor, STING [12,13,14,15]. In its inactive state, STING is anchored on the ER membrane as a dimer with the C-terminal domain exposed to the cytoplasm [16]. On binding cGAMP, STING dimers translocate to the Golgi and oligomerize where Tank-binding kinase 1 (TBK1) phosphorylates STING, and the activated TBK1 and Ikappa B kinase (IKK) further activate transcription factors IRF3 and NF-κB to produce type I IFN and proinflammatory cytokines [17,18,19]. STING can be directly activated by cyclic dinucleotides that are produced as second-messenger molecules in bacterial signal transduction such as cyclic diguanylic acid (c-di-GMP) and cyclic diadenylic acid (c-di-AMP), or cGAMP produced by cGAS in metazoan cells suggesting a widespread role in innate immunity [12].

The non-canonical STING pathway is activated by cellular stress stimuli such as nuclear DNA damage or mitochondrial DNA released into the cytoplasm caused by DNA damage agents and chemotherapy drugs. Recent studies also demonstrate non-canonical signaling in senescence, neurodegeneration, and autoimmune disorders by activating alternate signaling pathways [20,21,22,23,24,25,26,27,28,29,30,31]. For instance, Etoposide activated STING by IFI-16, and not cGAS, and recruited ATM, p53, and TRAF6 to transcriptionally activate NF-κB target genes and cytokines, rather than IRF3 [32]. In response to DNA damage agents, ionizing radiation, and release of mitochondrial DNA, STING altered transcriptional induction of genes regulating ROS homeostasis, which in turn controls subsequent DNA damage, providing feedback regulation [33]. Another study, in contrast, showed that cGAMP activates DNA damage repair (DDR) via STING-TBK1, but independent of IFN signaling [34]. In response to oxidized cholesterol, STING activated PERK to trigger epigenetic regulation by chromatin remodeling protein BRD4 and NF-κB promoting expression of genes in unique enhancer regions [35]. STING–PERK axis altered translation program in a non-canonical pathway attenuating kidney and lung fibrosis by regulating expression of a subset of proteins [36].

The activation of innate immune pathways is tightly coupled to cellular stress pathways. Stress stimuli activate the integrated stress response (ISR) signaling pathways to restore cellular homeostasis and converge on phosphorylation of the translation initiation factor, eIF2α, to inhibit translation [37]. The stalled translation preinitiation complexes containing mRNAs, initiation factors, small ribosomal subunits, RNA-binding proteins, together with the Ras-GAP SH3 domain-binding protein 1 (G3BP1) and T-cell-restricted intracellular antigen 1 (TIA1), coalesce into membrane-less phase separated foci forming stress granules (SGs) [38,39]. SG composition and proteins recruited vary depending on the type of stimulus and cell type but G3BP1 (Ras-GAP SH3 domain-binding protein 1) is required for nucleation in all contexts [40,41]. In addition to canonical SG, specialized antiviral SG (avSG) forms during viral infections to coordinate immune signaling [42,43,44]. The avSGs formed by dsRNA during RNA virus infection are unique and distinct, and we and others showed that they serve as a platform to enhance antiviral signaling and interferon (IFNβ) production by recruiting innate immune sensors and antiviral effectors to serve as a signaling hub [44,45,46,47]. Most studies have focused on dsRNA as a trigger for SG induction; whether and how SGs are induced and contribute to the innate signaling by dsDNA ligands is not known. As an ER-resident adaptor, STING participates in ER stress response mediated by ER-stress kinase, PERK, and participates in proteostatic mechanisms at the ER. In this study, we show that cytosolic DNA, exogenous and endogenous, activates the cGAS-STING pathway and PERK-mediated SG formation. STING is indispensable for activating this pathway leading to production of type I IFN and inflammatory cytokines. Inhibiting PERK by genetic ablation or pharmacological inhibition abrogates SG formation and innate signaling, an effect that is replicated in cells lacking key SG marker, G3BP1. DNA damage by Doxorubicin and Taxol activated ISR and SG formation, coupled with IFNβ and cytokine induction, while simultaneously inducing ROS and upregulating superoxide dismutase (SOD). Our results identify a previously unrecognized role of G3BP1 and stress granules induced by PERK in response to dsDNA as a novel mediator of cGAS-STING signaling that is relevant to an array of diseases like autoimmune disorders, cancer, fibrosis, and neurodegenerative disorder, in addition to the canonical innate immune signaling for pathogen evasion.

## 2. Materials and Methods

### 2.1. Chemicals, Reagents, and Antibodies

Chemicals, unless otherwise indicated, were from Sigma-Aldrich (St. Louis, MO, USA). Interferon stimulatory DNA (ISD), c-di-GMP (cDG), poly dA:dT, HT-DNA, HSV60, or 2′-3′cGAMP were from InvivoGen (San Diego, CA, USA). PERK inhibitor GSK2656157 was from Santa Cruz Biotechnology (Dallas, TX, USA). Hydrogen peroxide (H325-100) and puromycin (BP2956100) were purchased from Fisher Scientific (Hampton, NH, USA). Etoposide (E1383), Doxorubicin (D1515), Hydroxyurea (H8627), and Camptothecin (C9911) were from Sigma-Aldrich (St. Louis, MO, USA). IRE1 inhibitor (4μ8C, 14003-96-4) and Taxol (33069-62-4) were purchased from Cayman Chemicals (Ann Arbor, MI, USA). Poly I:C was from Calbiochem (San Diego, CA, USA). HA-STING plasmid was from Saurabh Chattopadhyay, University of Kentucky. Hanks’ balanced salt solution (HBSS) with calcium and magnesium, without phenol red (21-023-CV), was from Corning (Manassas, VA, USA). p-STING (50907), STING (13647), p-TBK1 (5482), TBK1 (3013), p-IRF3 (4947), IRF3 (4302), p-STAT1 (9167), STAT1 (9172), p-eIF2α (3398), eIF2α (5324), p-H2AX (9718), β-actin (3700), IFI16 (14970), DDX41 (15076), HA-Tag (3724), cleaved PARP (5625), cleaved Caspase 3 (9664), anti-mouse IgG, and anti-rabbit IgG horseradish peroxidase (HRP)-linked secondary antibodies were from Cell Signaling Technology (Danvers, MA, USA). G3BP1 (sc-81940) and TIA1 (sc-166247) were from Santa Cruz Biotechnology (Dallas, TX, USA). cGAS (26416-1-AP) and BiP (66574-1-Ig) were from Proteintech (Rosemont, IL, USA). siRNA towards DDX41 (sc-91765), IFI16 (sc-35633), and cGAS (sc-95512) were from Santa Cruz Biotechnology (Dallas, TX, USA). Enhanced chemiluminescence (ECL) reagents were from Boston Bioproducts (Ashland, MA, USA) and Bio-Rad (Hercules, CA, USA).

### 2.2. Cell Culture, Transfections, and Drug Treatments

The human fibrosarcoma cell line, HT1080 (a gift from Ganes Sen, Cleveland Clinic, Cleveland, OH, USA), G3BP1 KO [46], STING KO (this study), WT and *PERK*^−/−^ mouse embryonic fibroblasts (MEFs, a gift from Maria Hatzoglou, Case Western Reserve University, Cleveland, OH, USA), WT and *PKR*^−/−^ MEFs (a gift from Robert Silverman, Cleveland Clinic, Cleveland, OH, USA), HeLa cells (a gift from Robert Silverman, Cleveland Clinic, Cleveland, OH, USA), NuFF (human newborn foreskin fibroblast cells, a gift from Saurabh Chattopadhyay, University of Kentucky), and HEK293 (a gift from Fan Dong, University of Toledo) were cultured in Dulbecco’s modified minimal essential medium with 10% fetal bovine serum, 100 μg/mL penicillin/streptomycin, 2 mM L-glutamine, and nonessential amino acids. HT1080 stably expressing GFP-G3BP1 was maintained in Dulbecco’s modified minimal essential medium with 10% fetal bovine serum, 100 μg/mL penicillin/streptomycin, 2 mM L-glutamine, and nonessential amino acids supplemented with 500 μg/mL of G418 (Geneticin, Sigma-Aldrich (St. Louis, MO, USA)). Cells were maintained in 95% air and 5% CO_2_ at 37 °C. DNA ligands were transfected at indicated concentration as follows: interferon stimulatory DNA (ISD, 2 μg/mL), c-di-GMP (cDG, 5 μg/mL), poly dA:dT (5 μg/mL), HT-DNA (4 μg/mL), HSV60 (5 μg/mL), or 2′-3′cGAMP (5 μg/mL) using lipofectamine 2000 (Invitrogen, Carlsbad, CA, USA), according to the manufacturer’s protocol. Poly I:C (2 μg/mL) was transfected using PolyJet reagent (SignaGen Laboratories, Frederick, MD, USA), according to the manufacturer’s protocol. siRNA transfections (siRNA DDX41 (20 nM), siRNA IFI16 (20 nM), and siRNA cGAS (20 nM)) in HT1080 cells were performed using Lipofectamine 2000, according to manufacturer’s protocol (Invitrogen, CA, USA), and after 36 h, cells were transfected with the indicated DNA ligands and after 6 h, cell lysates were used for Western blotting and compared to mock control lysates. Mock controls included cells treated with transfection reagent alone. In experiments involving inhibitors, cells were preincubated with inhibitor GSK2656157 (5 μM) or IRE1 inhibitor (4μ8C, 2.5 μg/mL) or vehicle for mock control for 1 h prior to transfection and then replaced with growth medium. DNA-damaging agents Etoposide, Doxorubicin, Hydroxyurea, Camptothecin, and Taxol were diluted at indicated concentrations in prewarmed growth medium and added to cells.

### 2.3. Generation of Gene Knockout Cell Lines Using CRISPR/Cas9 System

The G3BP1 knockout in HT1080 cells has been described previously [46]. To generate STING knockout HT1080 cells using CRISPR/Cas9 gene editing system, TMEM173 double nickase plasmids (sc-403148-NIC, Santa Cruz Biotechnology) were used. Briefly, 1 × 10^5^ cells of HT1080 cells were plated into 1 well of a 24-well plate and transfected with 2 μg of plasmid using PolyJet reagent and after 36 h cells selected in 1 μg/mL puromycin and screened for GFP-positive clones. Puromycin-resistant cells were cloned by plating 2.5 cells/mL in a 48-well plate, and single-cell clones were screened and validated for gene editing by probing Westerns blots for absence of STING protein expression by immunoblotting.

### 2.4. Real-Time Stress-Granule Monitoring Assay

HT1080 cells stably expressing GFP-G3BP1 were seeded in a 12-well plate in triplicates and treated with inhibitors wherever indicated, followed by transfection with ligands or addition of DNA damage agents. Mock transfected cells not receiving DNA ligands or cells treated with vehicles used for DNA damage agents were used as control for non-specific stress effects. Cells were monitored for stress granule (SG) formation (represented as G3BP1 puncta) using EVOS M5000 fluorescence microscope (Thermo Scientific, Life Technologies, Carlsbad, CA, USA) at indicated times. Cells with *n* ≥ 5 G3BP1 puncta per cell were considered SG positive and included in analysis. The number of total and SG positive cells were quantified from at least 3 random fields from a minimum of 100 cells per treatment from three independent wells and plotted as percentage of SG positive cells (mean ± SD).

### 2.5. Immunofluorescence Analysis

Cells were cultured on glass coverslips, and after treatment, the cells were fixed with 4% paraformaldehyde (Boston Bioproducts, Ashland, MA, USA) for 15 min at room temperature, permeabilized with 0.2% Triton X-100 in phosphate-buffered saline (PBS) for 15 min, and blocked with 3% bovine serum albumin (BSA) and 0.02% Tween 20 for 1 h at room temperature for cells stained with G3BP1. For cells stained with p-H2AX, STING, BiP, and TIA1, cells were fixed in 100% pre-chilled methanol (Fisher Scientific) for 30 min at −20 °C, permeabilized with 0.5% Triton X-100 in phosphate-buffered saline (PBS) for 15 min, and blocked with 5% fetal bovine serum (FBS) and 0.2%Tween 20 for 1 h at room temperature. Cells were then washed with PBS and incubated overnight at 4 °C with indicated antibodies (G3BP1 -1:250, p-H2AX -1:400, STING -1:600, BiP -1:500, and TIA1 -1:250). Alexa 488 or Alexa 647-conjugated anti-immunoglobulin antibody (1:1500) (Molecular Probes, Eugene, OR, USA) was used as a secondary antibody. Cell nuclei were stained with Vectashield, with DAPI (4′,6-diamidino-2-phenylindole) (Vector Laboratories, Burlingame, CA, USA). Fluorescence and confocal microscopy assessments were performed with a Leica CS SP5 multiphoton laser-scanning confocal microscope (Leica Microsystems, Wetzlar, Germany) or Olympus IX81 Microscope (Olympus Corporation, Tokyo, Japan). All subsequent analysis and processing of images were performed using ImageJ software (v1.5) or Olympus Stream View software V2.3 (Olympus Corporation, Tokyo, Japan). To measure the intensity of p-H2AX, ROS foci, STING, and BiP foci, a line was drawn across the nucleus/foci and the intensities along the line were measured using a plot profile using ImageJ software. The arbitrary intensity was plotted according to an arbitrary distance for each cell.

### 2.6. RNA Isolation and Quantitative Real-Time PCR

Total RNA was isolated from cells plated in triplicate and treated with DNA ligands or DNA damage agents without or with inhibitors for indicated times using TRIzol reagent (Invitrogen), as per the manufacturer instructions. RNA was reverse-transcribed with random decamers and a RETROscript cDNA synthesis kit (Life Technologies; Thermo Fisher Scientific, Carlsbad, CA, USA). Quantitative reverse-transcription polymerase chain reaction (qRT-PCR) was performed using SYBR Green PCR Master Mix (Bio-Rad Laboratories Inc., Hercules, CA, USA) and gene-specific primers (Table 1), and the results were analyzed by the ΔCt or ΔΔCt method and normalized to *GAPDH* expression.

### 2.7. Western Blot Analysis

Immunoblot analysis of cell lysates was performed as previously described [48]. Briefly, cells treated as indicated were washed twice in cold phosphate-buffered saline (PBS) and lysed with Nonidet P-40 lysis buffer (0.5% NP-40, 90 mM KCl, 5 mM magnesium acetate, 20 mM Tris, pH 7.5, 5 mM β-mercaptoethanol, 0.1 M phenylmethylsulfonyl fluoride (PMSF), 0.2 mM sodium orthovanadate, 50 mM NaF, and 10 mM glycerophosphate), supplemented with protease inhibitor mixture (Roche Diagnostics GmbH, Mannheim, Germany), and incubated on ice for 20 min. The lysates were clarified by centrifugation at 12,000× *g* (4 °C for 15 min) and protein was quantified by Bradford assay (Bio-Rad Laboratories, CA, USA). Cell lysates (15–100 µg) were separated on SDS PAGE gels and proteins were transferred to nitrocellulose membranes (Bio-Rad, Hercules, CA, USA) or PVDF membrane (Bio-Rad, Hercules, CA, USA) and probed with antibodies according to the manufacturer’s recommendations. The immunoblots were developed using enhanced chemiluminescence reagents (Bio-Rad (Hercules, CA, USA) and Boston Bioproducts, Ashland, MA, USA). Images were processed using Adobe Photoshop CC v20.0.5 (Adobe, San Jose, CA, USA). In some instances, non-specific lanes were cropped to generate the images, and the boundaries are indicated in the representative figures. Representative immunoblots as indicated in the legends are shown. The density of protein bands on the immunoblots was quantified using Image J program.

### 2.8. IRF3 Nuclear Translocation Assay

HT1080 WT, G3BP1 KO, and STING KO cells plated in triplicate were transfected with GFP-IRF3 plasmid and 16 h later transfected with STING ligands or treated with Doxorubicin (0.8 μg/mL) or Taxol (100 nM). IRF3 nuclear translocation was monitored over 3–6 h (for ligand treatment) or over 24–36 h (for Doxorubicin and Taxol treatment), and images were captured on EVOS M5000 fluorescence microscope (Invitrogen, Life Technologies, Carlsbad, CA, USA). In every field, only cells where the GFP signal is nearly completely in the nucleus were counted as positive. These assays were reproduced using a blind approach with multiple researchers for cell counting to enhance experimental rigor and minimize subconscious bias. The total number of GFP-positive cells and cells showing nuclear translocation was quantified from random fields and plotted as percentage of GFP-IRF3 nuclear translocation. At least 3 independent fields from experiment performed in triplicate showing a minimum of 100 cells per treatment were analyzed for quantitation and data were plotted as percentage (mean ± SD).

### 2.9. ROS Detection Assay

HT1080 cells (8000 cells per well) were seeded in a 96-well plate and treated with Doxorubicin (0.8 μg/mL) 24 h later. After 36 h, supernatant media were aspirated carefully, and cells were washed twice with prewarmed 1× HBSS. Carboxy-H2DCFDA (C400, Thermo Fisher Scientific, Carlsbad, CA, USA) was added at 60 uL of 1 μM diluted in 1× HBSS and cells were incubated at 37 °C for 30–45 min and washed twice with HBSS. Cells were allowed to recover in 1× HBSS for 15 min, and fluorescence was detected using GFP channel on EVOS M5000 fluorescence microscope (Invitrogen, Life Technologies, Carlsbad, CA, USA). Total number of cells and cells showing green fluorescence were quantified from at least 3 independent fields and plotted as % ROS-positive cells. The GFP intensities of cells were estimated using ImageJ software (Version 1.54) (National Institutes of Health, Bethesda, MD, USA) and plotted as mean ± SD.

### 2.10. IFNβ Promoter Activation Reporter Assay

HT1080 WT and G3BP1 KO cells (1 × 10^5^) were seeded in a 12-well plate and transfected with plasmid expressing firefly luciferase gene under the control of IFNβ promoter (*IFNβ*-luc), along with pCH110 β-galactosidase expressing plasmid to normalize transfection efficiency and HA-STING plasmid. After 24 h, cells were transfected with pdA:dT (5 μg/mL) using lipofectamine 2000 and firefly luciferase activity was measured using luciferase assay reagents (Gold Biotechnology, St. Louis, MO, USA) and a luminescence plate reader (iD5 Molecular Devices, San Jose, CA, USA) and normalized to β-galactosidase levels. Data shown are normalized to controls receiving transfection reagent alone and empty vector and representative of three independent experiments performed in triplicate and shown as mean ± SE.

### 2.11. Cell Viability Assay

HT1080 WT, G3BP1 KO, and STING KO cells were mock-treated with vehicle or treated with Doxorubicin (0.8 μg/mL) or Taxol (100 nM) for indicated times and cell viability was determined by trypan blue exclusion assay. Briefly, 2.5 × 10^5^ cells per well were seeded in triplicates in 6-well plates and following treatment as above, cells were harvested and cell suspension was mixed 1:1 with 0.4% trypan blue solution (*w*/*v*) (Life Technologies, CA, USA) and viable cells were determined in three technical replicates using Countess 3 Automated Cell Counters (Life Technologies, Carlsbad, CA, USA) and normalized to mock-treated cells.

### 2.12. Statistical Analysis

Student’s *t*-tests were used for determining statistical significance of the results of comparisons between groups using Prism 8 (GraphPad, V8, Boston, MA, USA) software. The *p* values were obtained from a two-tailed, unpaired Student’s *t* test and are shown for all analyses in the relevant figures. *p* < 0.05 was considered significant in all cases. All values are presented as mean ± SEM from at least three independent experiments or are representative of three independent experiments performed in triplicate and shown as mean ± SD.

## 3. Results

### 3.1. Cytoplasmic dsDNA Induces Stress Granule Formation

**Figure 1:**
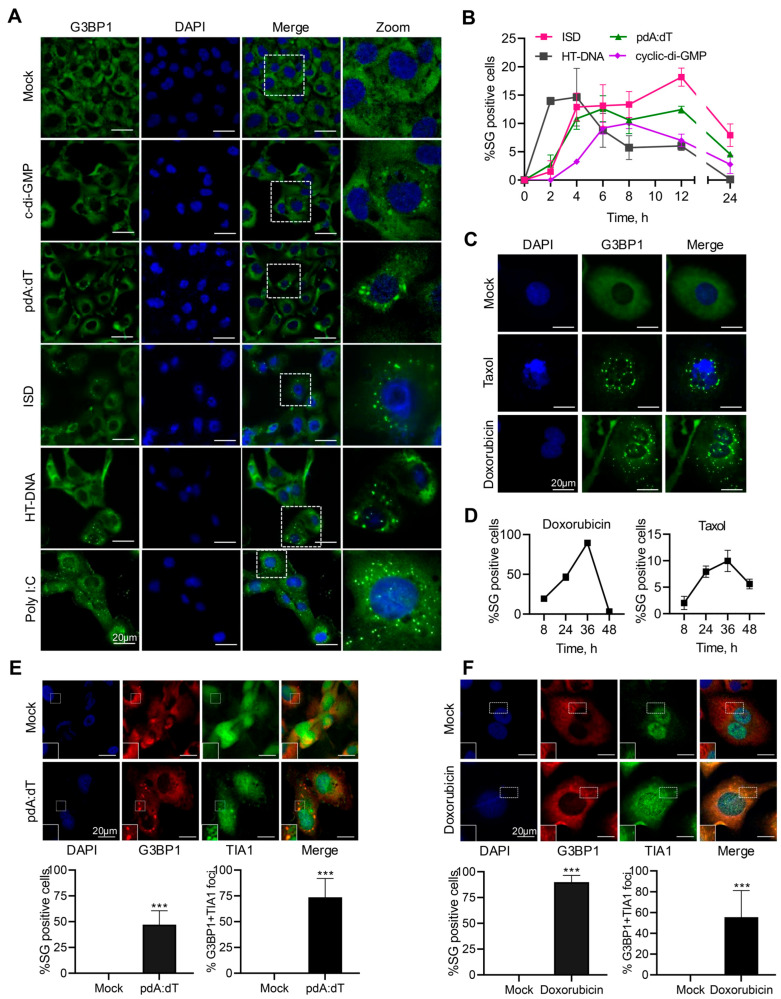
DNA ligands and DNA damage agents induce stress granule formation. HT1080 cells were mock-transfected or transfected with pdA:dT (5 μg/mL), cyclic-di-GMP (5 µg/mL), ISD (2 µg/mL), or HT-DNA (4 µg/mL) or synthetic dsRNA poly I:C (2 µg/mL), and 6 h later (**A**) SGs immunostained with anti-G3BP1 antibodies (green) and nuclei (DAPI, blue) and analyzed by confocal microscopy. (**B**) Kinetics of SG formation in HT1080 cells stably expressing GFP-G3BP1 treated as in (**A**) and % of cells showing GFP-G3BP1-positive foci were determined at indicated time points from at least 100 cells from three replicates. Results are mean ± SD. HT1080 cells were mock-treated or treated with Doxorubicin (0.8 µg/mL) or Taxol (100 nM) and (**C**) SGs immunostained with anti-G3BP1 antibodies (green) and nuclei (DAPI, blue) and analyzed by confocal microscopy. (**D**) Kinetics of SG formation in HT1080 cells stably expressing GFP-G3BP1 treated as in (**C**) and % of cells showing GFP-G3BP1-positive foci were determined at indicated time points from at least 100 cells from three replicates. Results are mean ± SD. HT1080 cells were transfected with pdA:dT (5 μg/mL) (**E**) or treated with Doxorubicin (0.8 µg/mL) (**F**) and cells were examined for the presence of the core SG marker G3BP1 (red) and SG marker TIA1 (green) and nuclei (DAPI, blue) by immunofluorescence staining and confocal microscopy. Percentage of cells showing G3BP1-positive SG puncta and % of cells that stained with both G3BP1 and TIA1 was determined from at least 100 cells from three replicates. Results are mean ± SD. The magnified images correspond to boxed regions. Scale bars, 20 µm. The results presented here are representative of at least three biological repeats. *** *p* < 0.001.

To determine if the anomalous presence of dsDNA in the cytoplasm induces stress granule formation, human fibrosarcoma cells, HT1080, were transfected with the following different types of immunostimulatory dsDNA ligands: (a) ISD (interferon stimulatory DNA), containing bacterial DNA motifs, (b) poly (pdA:dT, synthetic dsDNA), (c) HT-DNA (herring testis DNA), and (d) cDG (c-di-GMP, cyclic dinucleotide of bacterial origin), and localization of the key stress granule protein, G3BP1, in distinct stress granules was compared to mock-treated cells. For comparison with dsRNA ligands, cells were treated with synthetic dsRNA, polyI:C, previously shown to induce stress granules [46]. DNA ligands induced discrete G3BP1 puncta characteristic of stress granules similar to polyI:C, compared to diffuse G3BP1 distribution in mock-treated cells (Figure 1A). Stress granules (SGs) are phase-separated aggregates that assemble and disassemble in a dynamic way in response to stress stimuli [41,49]. To determine the dynamic nature of assembly/disassembly of SG, we monitored SG formation in real time in HT1080 cells stably expressing GFP-G3BP1 in response to the DNA ligands. The kinetics varied and we observed gradual increase over time in % cells showing SG assembly that declined over the course of recovery from stress (Figure 1B). Exposure of cells to DNA damage agents results in accumulation of cytoplasmic DNA, some in the form of micronuclei, triggering inflammatory response and cytokine production [20,50,51,52,53]. To determine if DNA damage agents induce formation of SG, Doxorubicin- or Taxol-treated HT1080 cells were analyzed for SG formation compared to mock-treated cells and SG formation in real-time was monitored in GFP-G3BP1 expressing cells. Both Doxorubicin and Taxol induced SG; however, compared to Taxol, Doxorubicin induced significantly more SG, and we have focused on Doxorubicin-induced pathways in these studies (Figure 1C,D). To characterize the canonical SG, HT1080 cells were transfected with pdA:dT or HT-DNA and stained for SG markers, G3BP1 and TIA1 and cells forming SG, and % of SG showing co-localization of both G3BP1 and TIA1 was determined by immunostaining and microscopy. We observed 70–80% of SG to show the presence of both G3BP1 and TIA1 as would be expected for canonical SG (Figure 1E). Doxorubicin-treated cells were stained for SG markers, G3BP1, and TIA1 and greater than 80% of SG show co-localization of both G3BP1 and TIA1 typical of SG (Figure 1F).

### 3.2. PERK Is Activated by Cytosolic dsDNA to Promote SG Formation

**Figure 2:**
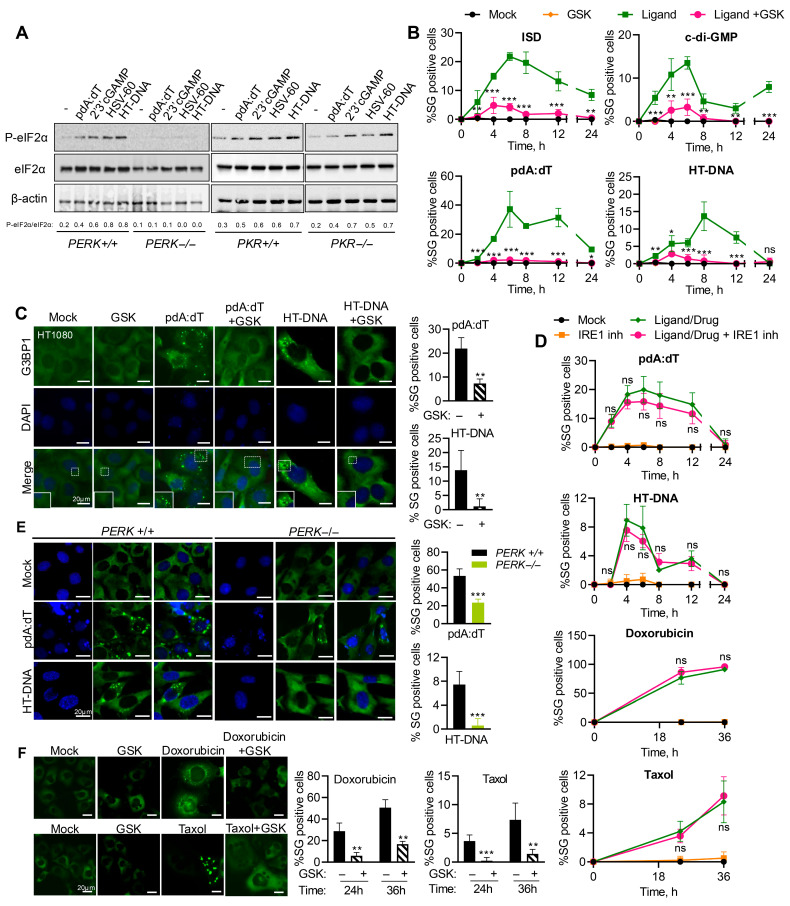
PERK activity is required for SG induction by DNA ligands and DNA damage agents. (**A**) WT, *PERK*^−/−^, or *PKR*^−/−^ MEFs were transfected with pdA:dT (5 μg/mL), 2′-3′-cGAMP (5 μg/mL), HSV60 (5 µg/mL), or HT-DNA (4 µg/mL) for 6 h, and phosphorylation of eIF2α was determined in immunoblots compared to total eIF2α and normalized to β-actin. The ratios of p-eIF2α/total eIF2α were quantified by Image J. (**B**) Kinetics of SG formation in HT1080 cells stably expressing GFP-G3BP1 treated with indicated DNA ligands without or with PERK inhibitor (GSK2656157, 5 µM) and % of cells showing GFP-G3BP1-positive foci were determined at indicated time points from at least 100 cells from three replicates. Results are mean ± SD. (**C**) HT1080 and (**E**) *PERK*^+/+^ and *PERK*^−/−^ MEFs treated with pdA:dT (5 μg/mL) or HT-DNA (4 μg/mL), without or with PERK inhibitor (GSK, 5 µM) and SG immunostained with anti-G3BP1 (green) and nuclei (DAPI, blue) and SG positive cells were analyzed by confocal microscopy and quantitated. Percentage of cells showing G3BP1-positive SG puncta was determined from at least 100 cells from three replicates. Results are mean ± SD. (**D**) Kinetics of SG formation in HT1080 GFP-G3BP1 expressing cells treated with indicated treatments without or with IRE1 inhibitor and % of cells showing GFP-G3BP1-positive foci were determined at indicated time points from at least 100 cells from three replicates. Results are mean ± SD. (**F**) HT1080 cells stably expressing GFP-G3BP1 treated with Doxorubicin (0.8 µg/mL) or Taxol (100 nM) with or without PERK inhibitor (GSK) and SG formation quantified from at least 100 cells from three replicates. Results are mean ± SD. The magnified images correspond to boxed regions. Scale bars, 20 µm. The results presented here are representative of at least three biological repeats. * *p* < 0.05; ** *p* < 0.01; and *** *p* < 0.001; ns: not significant.

Of the stress kinases, PKR and PERK are activated by dsRNA or ER-stress and phosphorylate an eukaryotic translation initiation factor, eIF2α, to inhibit translation, which nucleates the SG formation [37]. To determine the stress kinase that transmits the dsDNA signals, WT, *PERK*^−/−^, and *PKR*^−/−^ mouse embryonic fibroblasts (MEFs) were transfected with pdA:dT, HT-DNA, HSV-60, a 60 bp viral DNA motif derived from Herpes Simplex Virus-1, or 2′-3′cGAMP (STING activator), and phosphorylation of eIF2α (p-eIF2α) in cell lysates was compared by immunoblotting. MEFs lacking PERK showed no eIF2α phosphorylation in response to any of the DNA ligands compared to WT MEFs, while MEFs lacking PKR showed p-eIF2α like WT cells (Figure 2A). WT, *PERK*^−/−^, and *PKR*^−/−^ MEFs were treated with polyI:C or 2′-3′cGAMP and SG formation was monitored by staining G3BP1 by immunofluorescence microscopy. MEFs lacking PKR were defective in polyI:C-induced SG formation but not with cGAMP, and PERK lacking MEFs was proficient in polyI:C-induced SG but defective in cGAMP-induced SG formation, confirming the specificities of the stress kinases (Figure S1). In HT1080 cells, at all times in the course of SG induction by pdA:dT, cDG, HT-DNA, and ISD ligands, inhibition of PERK activity with inhibitor GSK2656157 (GSK) resulted in significant reduction or complete inhibition of SG formation (Figure 2B). We performed immunofluorescence assays to detect G3BP1 in SG formed by cells pretreated with GSK or mock-treated (Figure 2C). Inhibiting PERK activity with GSK inhibitor significantly reduced G3BP1 puncta in response to both ligands. We observed similar induction of SG in primary NuFF cells (human newborn foreskin fibroblasts) transfected with pdA:dT, which was inhibited by GSK pretreatment, extending the relevance of our observations to primary cells (Figure S2). Cells treated with IRE1 inhibitor showed similar induction of SG by pdA:dT, HT-DNA, Doxorubicin, or Taxol during the time course of treatment (Figure 2D). The requirement of PERK activity for SG induction by transfection of these dsDNA ligands was further confirmed in *PERK*^−/−^ MEFs compared to WT MEFs (Figure 2E). To test if endogenous dsDNA produced by DNA damage agents also requires PERK activity to induce SG, HT1080 cells stably expressing GFP-G3BP1 were mock-treated or pretreated with GSK inhibitor in cells exposed to Doxorubicin or Taxol. As with the DNA ligands transfected to cells, inducing DNA damage resulted in SG formation, which could be significantly reduced by inhibiting PERK activity (Figure 2F). Taken together, our results show that cytoplasmic presence of dsDNA, introduced ectopically or produced from endogenous sources, can activate stress response pathways, specifically PERK, to phosphorylate eIF2α-promoting SG formation.

### 3.3. STING Is Required for Signaling and SG Formation by dsDNA Ligands

**Figure 3:**
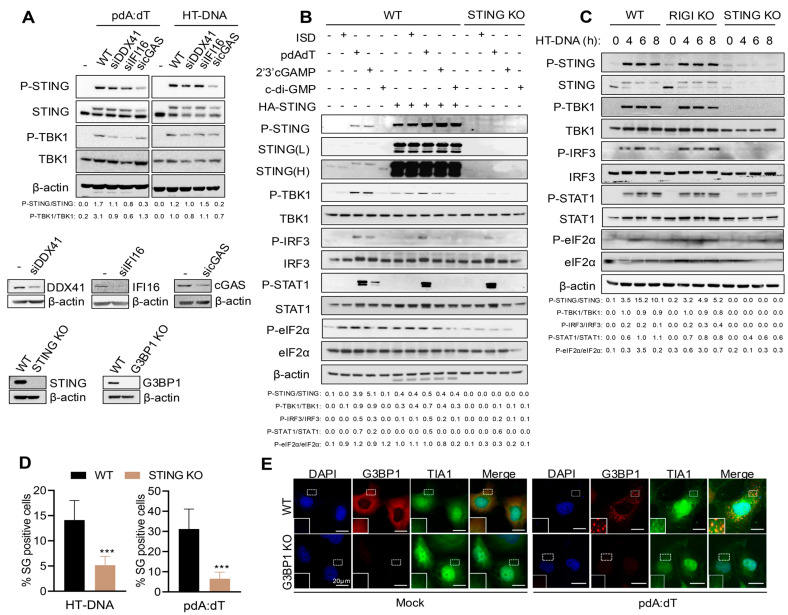
The cGAS-STING signaling pathway is required for SG induction by DNA ligands. (**A**) HT1080 cells were treated with siRNA for 36 h to knockdown DDX41, IFI16, or cGAS, followed by transfection with pdA:dT (5 μg/mL) or HT-DNA (4 μg/mL) for 6 h, and cell lysates were analyzed for phosphorylation of STING and TBK1 compared to total STING and total TBK1 normalized to β-actin levels. The ratios of p-STING/total STING and p-TBK1/total TBK1 were quantified by Image J. The efficiency of knockdown by siRNA was compared to control cells and determined by immunoblot analysis. (**B**) WT, cells expressing HA-STING, or STING KO HT1080 cells. (**C**) WT, RIG-I KO, and STING KO HT1080 cells were transfected with the indicated STING ligands for 8 h or indicated times and cell lysates were probed with indicated antibodies on immunoblots; two exposures of STING (L, low and H, high) of the same blot are shown. The ratios of the indicated phospho-proteins/total proteins for the antibodies probed were quantified by Image J. (**D**) WT and STING KO HT1080 cells transfected with pdA:dT (5 μg/mL) or HT-DNA (4 μg/mL) and SG were immunostained with anti-G3BP1, and SG positive cells were quantitated. Percentage of cells showing G3BP1-positive SG puncta was determined from at least 100 cells from three replicates. Results are mean ± SD. (**E**) WT and G3BP1KO HT1080 cells were transfected with pdA:dT (5 μg/mL) or mock-transfected and after 8 h were immunostained for SG markers G3BP1 (red) and TIA1 (green) and nuclei (DAPI, blue), and SG positive cells were analyzed by confocal microscopy. The magnified images correspond to boxed regions. Expression of STING or G3BP1 in KO cells was compared to WT cells by immunoblot analysis. Scale bars, 20 µm. The results presented here are representative of at least three biological repeats. *** *p* < 0.001.

Multiple cytosolic DNA sensors like cyclic GMP-AMP synthase (cGAS), interferon gamma-inducible 16 (IFI16), absent in melanoma 2 (AIM2), DEAD-Box Helicase 41 (DDX41), and Z-DNA-binding protein (ZBP1) are expressed in a cell-type and context-specific manner to detect dsDNA [5,6,7,8]. Based on the literature, cGAS is the primary sensor, and upon binding endogenous or exogenous dsDNA, it synthesizes a second messenger molecule 2′-3′-cyclic GMP-AMP (cGAMP), which binds to stimulator of IFN genes (STING) on endoplasmic reticulum (ER) and triggers downstream signaling to produce type I interferons (IFNs) and proinflammatory cytokines to activate immune response [10,12]. To determine the DNA sensors that relay this signal in HT1080 cells, we used siRNA to knockdown cGAS, DDX41, or IFI16 expression and determined activation of STING and TBK1 by phosphorylation on immunoblots (Figure 3A). Cells with cGAS KD (knockdown) showed reduced phosphorylation of STING and TBK1 with pdA:dT and HT-DNA. Based on these results, in this study, we focused on comparing signaling events mediated by STING in response to pdA:dT and HT-DNA using CRISPR/Cas9 STING KO cells. Additionally, overexpression of STING activates and induces IFNβ production and signaling pathway [3]. We compared the activation of signaling pathways leading to IFNβ production in WT, WT cells expressing HA-STING and STING KO activated with the DNA ligands and cyclic dinucleotides (Figure 3B,C). To rule out the role of RNA sensors, we analyzed the signaling pathway activated by HT-DNA in WT, STING KO, and RIG-I KO cells (Figure 3C). As demonstrated in the literature, compared to WT cells, HA-STING expression resulted in enhanced ligand-induced phosphorylation of STING and TBK1 that is required for transcriptional induction of IFNβ. Secreted IFNβ via IFNAR receptor activates JAK-STAT1 signaling pathway and we observed p-STAT1 in response to DNA ligands. Consistent with our previous observation, phosphorylation of eIF2α, indicative of activation of integrated stress response, was seen in ligand-treated samples and in HA-STING lysates. Cells lacking STING showed dampened activation of IFNβ signaling and p-eIF2α, demonstrating an integral role of STING in mediating ligand-activated stress response. We then compared SG formation by pdA:dT and HT-DNA in WT and STING KO cells. As expected for cells defective in p-eIF2α, fewer SG positive cells were observed in STING KO cells compared to WT cells (Figure 3D). We further used CRISPR/Cas9 KO G3BP1 KO cells and stained for another SG marker, TIA1 in cells transfected with pdA:dT. WT cells showed G3BP1 and TIA1 localizing to SG, and in cells lacking G3BP1, we confirmed that SGs, were not formed as no TIA1 positive puncta were observed (Figure 3E). Our results demonstrate the role of cGAS as a DNA sensor signaling through ER-resident protein STING to activate stress response pathway and IFNβ production to promote SG formation and trigger IFNβ signaling cascade.

### 3.4. DNA Damage Signaling Mediated by STING and G3BP1 Activates eIF2α and SG Formation

**Figure 4:**
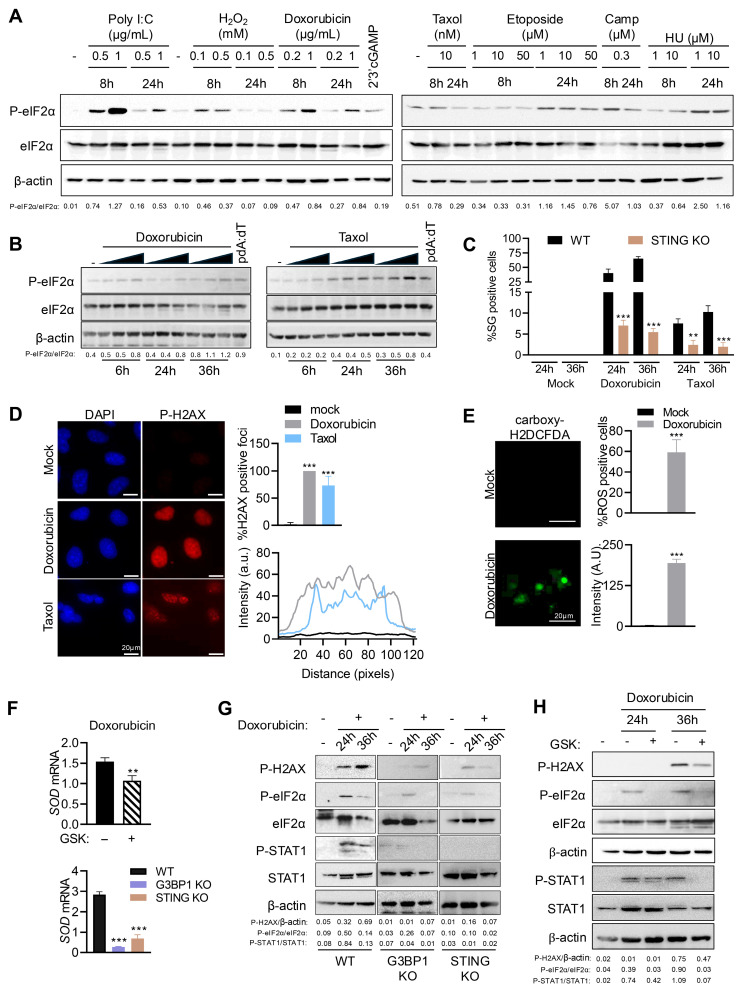
DNA damage agents signal via STING and G3BP1 to activate ISR and SG formation. HT1080 cells were treated with (**A**) varying amounts of DNA damage agents as indicated or transfected with polyI:C (2 μg/mL) for indicated times, (**B**) increasing concentration of Doxorubicin or Taxol for times as indicated, and phosphorylation of eIF2α in cell lysates was determined in immunoblots and compared to total eIF2α and normalized to β-actin. The ratios of p-eIF2α/total eIF2α were quantified by Image J. (**C**) WT and STING KO HT1080 cells treated with Doxorubicin (0.8 µg/mL) or Taxol (100 nM) for 24 h or 36 h, and SGs immunostained with anti-G3BP1 and SG positive cells were quantitated. Percentage of cells showing G3BP1-positive SG puncta was determined from at least 100 cells from three replicates. Results are mean ± SD. (**D**) Representative images and quantification of Doxorubicin- or Taxol-induced p-H2AX (γH2AX) foci in HeLa cells were analyzed by immunostaining and confocal microscopy. Percentage of cells showing p-H2AX foci and intensities of p-H2AX foci were quantified using ImageJ. (**E**) HT1080 cells were treated with Doxorubicin (0.8 µg/mL) for 36 h and stained for ROS analysis with carboxy-H2DCFDA (1 µM) for 45 min and imaged by fluorescence microscopy. Representative images and quantification of fluorescence of carboxy-H2DCFDA (1 µM) were determined as % of cells showing fluorescent foci (green), and intensities of the foci were quantified using ImageJ. For (**D**,**E**), at least 100 cells from three replicates were used in analysis and the results are shown as mean ± SD. (**F**) HT1080 cells plated in three independent wells were treated with Doxorubicin (0.8 µg/mL) without or with PERK inhibitor (GSK), or WT, G3BP1 KO, and STING KO HT1080 cells plated in three independent wells were treated with Doxorubicin (0.8 µg/mL) for 36 h and mRNA levels of *SOD* were measured by qRT-PCR and normalized to *GAPDH* mRNA levels. Data represent mean ± SE from three independent experiments. (**G**) WT, G3BP1 KO, and STING KO HT1080 cells were treated with Doxorubicin (0.8 µg/mL) for indicated times, (**H**) HT1080 cells were treated with Doxorubicin without or with PERK inhibitor (GSK) for indicated times, and cell lysates were probed with indicated antibodies. The ratios of p-eIF2α/total eIF2α and p-STAT1/total STAT1 were quantified by Image J. Scale bars, 20 µm. The results presented here are representative of at least three biological repeats. ** *p* < 0.01; and *** *p* < 0.001.

DNA damaging agents such as topoisomerase inhibitors promote nuclear DNA damage, causing accumulation of dsDNA in the cytoplasm that induces type I IFN and cytokine expression [20,51]. We treated HT1080 cells with varying concentrations of indicated chemotherapeutic drugs, H_2_O_2_ or polyI:C for 8 h or 24 h, or cGAMP (8 h), and analyzed phosphorylation of eIF2α in cell lysates. Interestingly, all DNA damage agents activated stress response pathway inducing eIF2α phosphorylation, albeit to different extents, suggesting a likely common mechanism induced by accumulation of dsDNAs (Figure 4A). We demonstrated the activation of eIF2α in response to increasing concentration of Doxorubicin or Taxol on immunoblots at 6, 24, or 36 h and compared with pdA:dT (Figure 4B). In line with the activation of eIF2α, we observed increase in SG formation, with Doxorubicin inducing more SG than Taxol (Figure 4C and Figure S3). More importantly, as with DNA ligands, STING KO cells showed three-fold less SG than WT cells treated with either DNA damage agents. DNA damage agents trigger H2AX phosphorylation, γH2AX, which corresponds to dsDNA breaks, and the intensity of the response is proportional to the number and size of double-strand break foci [54]. Damaged DNA fragments that enter the cytosol can be sensed by cGAS/STING further activating downstream signaling processes [20]. In addition to its role in DNA sensing, STING also regulates ROS levels through non-canonical signaling, so we characterized the DNA damage response (DDR)-regulated ROS activity [33]. We found that in Doxorubicin-treated cells nearly 100% cells stained positive for γH2AX foci, compared to 73% of Taxol-treated cells with consistent greater intensity in Doxorubicin compared to Taxol (Figure 4D). Using Carboxy-H2DCFDA as an indicator of cellular ROS, we found that 59% of Doxorubicin-treated cells showed an increase in fluorescence, indicating high ROS levels in the cells that correlate with DNA damage (Figure 4E). For subsequent analysis of DDR-induced STING activation, we used Doxorubicin as the activator of endogenous DNA sensing response. To determine the contribution of STING and G3BP1, and by extension of SG induced, to ROS homeostasis in response to Doxorubicin, WT, STING KO, and G3BP1 KO cells were treated with Doxorubicin, and mRNA levels of *MnSOD*, an antioxidant enzyme that counters reactive oxygen produced in cells, were monitored. Doxorubicin induced transcription of *MnSOD* in WT cells while STING KO and G3BP1 KO showed 3- to 4-fold decrease in mRNA levels (Figure 4F). To further confirm if PERK activity was required for this effect, cells were pretreated with PERK inhibitor GSK in the presence of Doxorubicin and we observed a statistically significant decrease in *MnSOD* mRNA levels compared to mock-treated cells (Figure 4F). To directly demonstrate the role of STING and G3BP1 in the DNA damage signaling pathway and stress response, lysates of WT, G3BP1 KO, and STING KO cells treated with doxorubicin for 24 or 36 h were analyzed for phosphorylation of H2AX (γH2AX), eIF2α, and STAT1. In WT cells, Doxorubicin promoted γH2AX, p-eIF2α, and p-STAT1, which was significantly abrogated in G3BP1 KO and STING KO cells (Figure 4G). Inhibiting PERK activity with GSK inhibitor in Doxorubicin-treated WT cells reduced γH2AX, p-eIF2α, and p-STAT1, consistent with our findings that activation of the STING pathway also activated the stress kinase PERK activity, triggering SG formation and IFNβ signaling (Figure 4H). As chemotherapeutic drugs, Doxorubicin and Taxol cause DNA damage, generate ROS to promote cellular stress, and induce programmed cell death; treatment with Doxorubicin or Taxol induced cell death which increased with time, as estimated by trypan blue dye exclusion assay (Figure S4A). Compared to WT cells, G3BP1 or STING KO cells showed greater cell viability with Doxorubicin and Taxol, and cell lysates showed reduced cleavage of PARP and Caspase-3, which are hallmarks of apoptosis (Figure S4B,C). One of the mechanisms of Doxorubicin-induced apoptosis is through upregulation of proapoptotic proteins like Noxa [55]. We observed induction of Noxa mRNA levels in WT cells with doxorubicin, which was abrogated in G3BP1 or STING KO cells (Figure S4D). These results support the role of dsDNA signaling in STING and G3BP1-mediated DNA damage response that can promote stress response by ROS and eventually induce apoptosis.

### 3.5. Activation of Stress Response by DNA Ligands Promotes IFN Signaling Involving G3BP1 and STING

**Figure 5:**
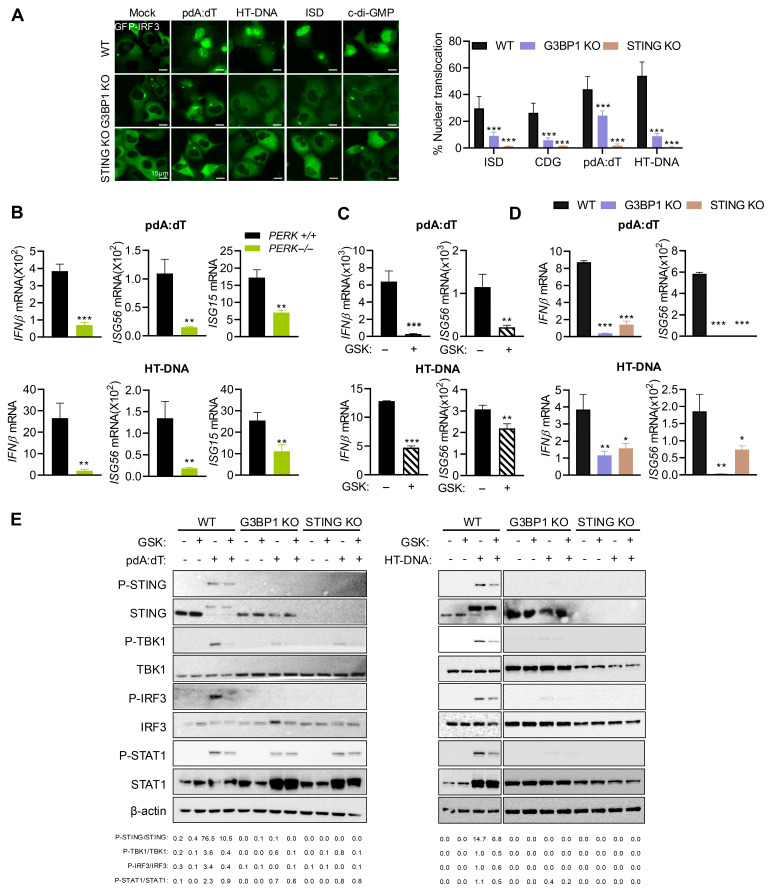
PERK activation by DNA ligands promotes IFN signaling involving G3BP1 and STING. (**A**) WT, STING KO, and G3BP1 KO cells plated in triplicate wells were transfected with GFP-IRF3, and 16 h later, the cells were treated with the indicated ligands or mock-treated and imaged after 6 h using fluorescence microscope. Total number of GFP positive cells and cells showing nuclear translocation was quantified from random fields and plotted as percentage of GFP-IRF3 nuclear translocation. At least 3 independent fields from experiment performed in triplicate showing a minimum of 100 cells per treatment were analyzed for quantitation and data were plotted as percentage (mean± SD). Representative images are shown. Scale bars, 15 µm. (**B**) *PERK*^+/+^ and *PERK*^−/−^ MEFs, plated in three independent wells, were transfected with pdA:dT (5 μg/mL) or HT-DNA (4 μg/mL) and 6 h later mRNA levels of *IFNβ*, *ISG56,* and *ISG15* were measured by qRT-PCR and normalized to *GAPDH* mRNA levels and plotted as fold induction. Data represent mean ± SE from three independent experiments. (**C**) HT1080 cells were transfected with pdA:dT or HT-DNA without or with PERK inhibitor (GSK). (**D**) WT, G3BP1 KO, or STING KO HT1080 cells plated in three independent wells were transfected with pdA:dT (5 μg/mL) or HT-DNA (4 μg/mL), and 6 h later mRNA levels of *IFNβ* and *ISG56* were measured by qRT-PCR and normalized to *GAPDH* mRNA levels and plotted as fold induction. Data represent mean ± SE from three independent experiments. (**E**) WT, G3BP1 KO, or STING KO HT1080 cells were transfected with pdA:dT (5 μg/mL) or HT-DNA (4 μg/mL) without or with PERK inhibitor (GSK), and 8 h later cell lysates were probed with indicated antibodies on immunoblots. The ratios of the indicated phospho-proteins/total proteins for the antibodies probed were quantified by Image J. The results presented here are representative of at least three biological repeats. * *p* < 0.05; ** *p* < 0.01; and *** *p* < 0.001.

Cytosolic DNA, including endogenous DNA released by DNA damage, is sensed by cGAS to produce cGAMP that binds and activates STING, which translocates from ER to Golgi, resulting in phosphorylation of TBK1 and IRF3. STING also activates NF-κB; both IRF3 and NF-κB subsequently translocate to the nucleus to transcriptionally induce type I IFN and inflammatory cytokines [12]. Our results show an important role of PERK in signaling by dsDNA ligands and SG formation, and our previous studies showed an important role of antiviral stress granules induced by dsRNA as a platform to integrate IFNβ production and signaling [46]. We first determined the involvement of IRF3 in dsDNA ligand-mediated IFNβ signaling in WT, STING KO, and G3BP1 KO cells. The translocation of GFP-IRF3 to the nucleus in cells transfected with pdA:dT, HT-DNA, ISD, or cDG was monitored, and STING KO and G3BP1 KO cells showed 3- to 5-fold decrease in nuclear IRF3 translocation compared to WT cells. Notably, the lack of STING had a greater impact on IRF3 nuclear translocation (Figure 5A). To examine the role of stress response and PERK activity on IRF3-mediated IFNβ signaling and induction of IFN-stimulated genes (ISGs), WT and *PERK*^−/−^ MEFs were transfected with pdA:dT or HT-DNA, and mRNA levels of *IFNβ*, *ISG56*/*P56*/*IFIT1*, and *ISG15* were compared. In line with reduced IRF3 nuclear translocation, mRNA levels of *IFNβ* and ISGs were reduced in cells lacking PERK in response to DNA ligands (Figure 5B). Inhibiting PERK activity with inhibitor GSK in HT1080 cells showed a similar decrease in *IFNβ* and *ISG56* mRNA levels, following transfection with pdA:dT or HT-DNA (Figure 5C). To test the importance of STING and G3BP1 as mediators of dsDNA-activated IFN signaling and response, we transfected WT, STING KO, and G3BP1 KO cells with pdA:dT or HT-DNA, compared mRNA levels of *IFNβ* and *ISG56* by RT-PCR, and analyzed cell lysates on immunoblots for activation of innate signaling proteins. As expected, knockout of STING and G3BP1 in HT1080 cells significantly reduced transcriptional induction of *IFNβ* and consequently *ISG56* (Figure 5D). Consistent with this observation, DNA ligand-induced phosphorylation of STING, TBK1, IRF3, and STAT1 was impaired in G3BP1 KO and STING KO. Importantly, treatment with GSK inhibitor decreased phosphorylation of these proteins in the signaling cascade leading to IFNβ production (Figure 5E). Collectively, these results show that dsDNA ligands activate innate signaling and stress response pathway and together regulate IFN production and signaling.

**Figure 6:**
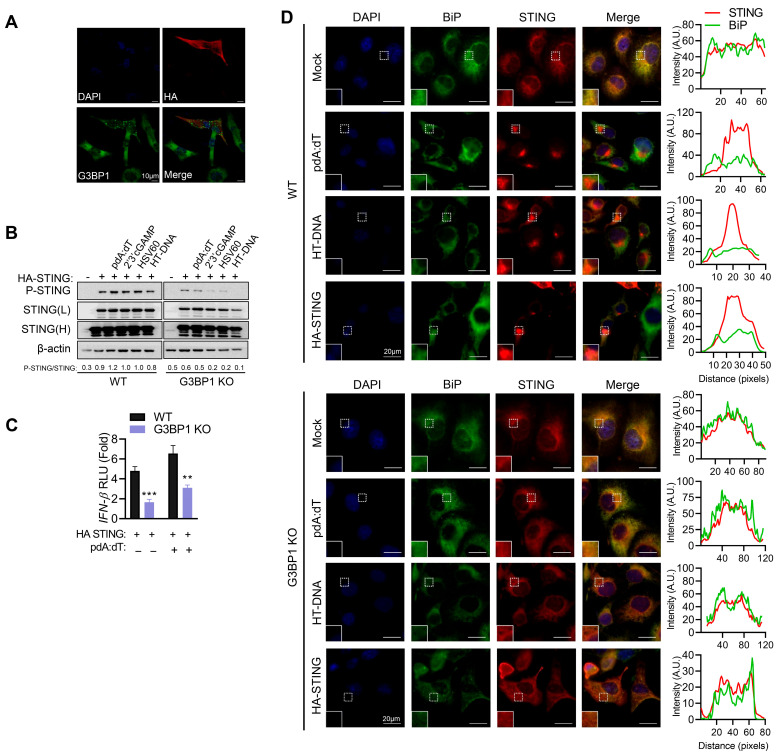
G3BP1 regulates STING activity. (**A**) HEK293 cells transfected with HA-STING plasmid and 24 h later fixed and immunostained with anti-G3BP1 and anti-HA antibody and analyzed by confocal microscopy. Representative images of cells from two biological repeats are shown. Scale bar 10 µm. (**B**) HT1080 WT and G3BP1 KO cells mock-transfected or transfected with HA-STING plasmid and 24 h later transfected with pdA:dT (5 μg/mL), 2′-3′-cGAMP (5 μg/mL), HSV60 (5 µg/mL), or HT-DNA (4 µg/mL), and after 8 h cell lysates were probed with p-STING and STING antibodies on immunoblots; two exposures of STING (L, low and H, high) of the same blot are shown. The ratios of the p-STING/total STING were quantified by Image J. (**C**) WT and G3BP1 KO cells were plated in three independent wells and transfected with HA-STING or vector and *IFNβ*-luc plasmid along with β-galactosidase plasmid and 24 h later mock-transfected or transfected with pdA:dT (5 μg/mL) and after 8 h luciferase activity was measured and normalized to β-galactosidase levels. Data shown are normalized to controls receiving transfection reagent alone and empty vector and representative of three independent experiments performed in triplicate and shown as mean ± SE. (**D**) HT1080 WT and G3BP1 KO cells plated on coverslips and 24 h later were transfected with pdA:dT (5 μg/mL) or HT-DNA (4 μg/mL) and fixed after 6 h. One set of coverslips were transfected with HA-STING plasmid and fixed after 24 h. Cells were stained with anti-STING (red) and anti-BiP (green), an ER marker, and nuclei (DAPI, blue), and analyzed by confocal microscopy. The magnified images correspond to boxed regions and line scans used to assess colocalization (separate colors shown on graphics) of markers. Scale bars, 20 µm. The results presented here are representative of at least three biological repeats or as noted. ** *p* < 0.01; and *** *p* < 0.001.

To understand the mechanistic relationship between STING and G3BP1, we overexpressed HA-STING in HEK293 cells and analyzed G3BP1 by immunofluorescence microscopy. HA-STING expression activates STING signaling and we observed G3BP1 puncta characteristic of SG only in cells expressing HA-STING. More importantly, G3BP1 and STING did not co-localize, ruling out regulation by direct interaction (Figure 6A). We analyzed activation of STING signaling in cell lysates of WT and G3BP1 KO cells overexpressing HA-STING or not and treated with various DNA ligands. Phosphorylation of STING was significantly reduced in cells lacking G3BP1, compared to WT cells (Figure 6B). Signaling to IFN promoter by over-expression of STING, which is shown in the literature to activate IFN production independent of ligand [3], was significantly reduced in G3BP1 KO cells, compared to WT cells in the absence of ligand stimulation. Treatment with pdA:dT increased IFN promoter activity in WT cells and the decrease in promoter induction in G3BP1 KO cells remained significant (Figure 6C). To address if G3BP1 alters STING trafficking, we treated WT and G3BP1 KO cells with pdA:dT or HT-DNA and checked localization of STING with ER marker BiP by immunofluorescence microscopy (Figure 6D). In unstimulated cells, STING co-localizes with BiP in WT cells, and on treatment with DNA ligands, STING translocation to ERGIC compartment and aggregation as speckles is observed as loss of co-localization with BiP and appearance of STING aggregates. In G3BP1 KO cells, we do not see STING translocation or aggregation when treated with DNA ligands, and STING continues to remain in ER, as evident from co-localization with BiP. Interestingly, in cells overexpressing HA-STING without ligand stimulation, we see STING aggregates which are not as distinct as in cells treated with ligands, and in G3BP1 KO cells STING appears more diffuse with ER staining. We have not shown co-localization with ERGIC markers but infer trafficking out of ER from the loss of ER localization with BiP. Our data suggest that over-expression of STING as shown in the literature activates signaling to IFN, and our results show it does not bypass the need for G3BP1. We infer from these data that G3BP1 likely alters STING trafficking; a more thorough study to fully dissect the mechanisms involved in the various steps in the pathways is needed.

### 3.6. Activation of PERK and Stress Granules Is Required for Cytokine Induction by DNA Ligands and Doxorubicin

**Figure 7:**
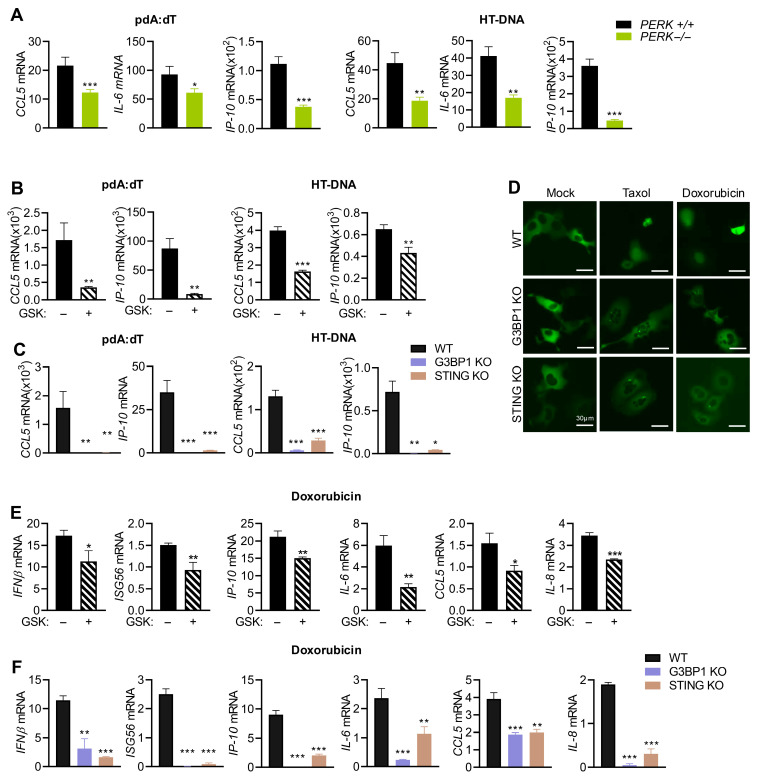
DNA ligands and DNA damage agents transcriptionally induce cytokines mediated by STING, G3BP1, and PERK activity. (**A**) *PERK*^+/+^ and *PERK*^−/−^ MEFs plated in three independent wells were transfected with pdA:dT (5 μg/mL) or HT-DNA (4 μg/mL), and after 6 h mRNA levels of *CCL5*, *IL-6*, and *IP-10* were measured by qRT-PCR and normalized to *GAPDH* mRNA levels and plotted as fold induction. Data represent mean ± SE from three independent experiments. (**B**) HT1080 cells plated in three independent wells were transfected with pdA:dT (5 μg/mL) or HT-DNA (4 μg/mL) without or with PERK inhibitor (GSK); after 6 h, mRNA levels of *CCL5* and *IP-10* were measured by qRT-PCR and normalized to *GAPDH* mRNA levels and plotted as fold induction. (**C**) WT, G3BP1 KO, or STING KO HT1080 cells plated in three independent wells were transfected with pdA:dT (5 μg/mL) or HT-DNA (4 μg/mL), and after 6 h mRNA levels of *CCL5* and *IP-10* were measured by qRT-PCR and normalized to *GAPDH* mRNA levels and plotted as fold induction. Data represent mean ± SE from three independent experiments. (**D**) WT, G3BP1 KO, and STING KO HT1080 cells plated in triplicate were transfected with GFP-IRF3, and 16 h later, the cells were treated with Doxorubicin (0.8 µg/mL) or Taxol (100 nM) or mock-treated and imaged using fluorescence microscope. Total number of GFP-positive cells and cells showing nuclear translocation was quantified from random fields and plotted as percentage of GFP-IRF3 nuclear translocation. At least 3 independent fields from experiment performed in triplicate showing a minimum of 100 cells per treatment were analyzed for quantitation and data were plotted as percentage (mean± SD). Representative images are shown. Scale bar, 30 µm. (**E**) HT1080 cells plated in three independent wells were treated with Doxorubicin (0.8 µg/mL) without or with PERK inhibitor (GSK). (**F**) WT, G3BP1 KO, or STING KO HT1080 cells plated in three independent wells were treated with Doxorubicin (0.8 µg/mL), and after 36 h mRNA levels of *IFNβ*, *ISG56*, *IP-10*, *IL-6*, *CCL5*, and *IL-8* were measured by qRT-PCR and normalized to *GAPDH* mRNA levels and plotted as fold induction. Data represent mean ± SE from three independent experiments. * *p* < 0.05; ** *p* < 0.01; and *** *p* < 0.001.

We have so far shown that cytosolic DNA signals via STING activate PERK-mediated stress response to induce SG and both STING and G3BP1 are required for IFNβ signaling. DNA damage agents promote γH2AX, ROS production, and signal via STING and G3BP1 to promote SG formation by PERK. We next sought to determine if PERK activity was also required for cytokine induction by DNA ligands. WT and *PERK*^−/−^ MEFs were treated with pdA:dT or HT-DNA, and mRNA levels of *CCL5*, *IP-10*, and *IL-6* were analyzed. As with *IFNβ* and ISG induction, mRNA levels of cytokines were also significantly lower in cells lacking PERK compared to WT cells (Figure 7A). The results could be extended to HT1080 cells, wherein inhibiting PERK by GSK resulted in 2- to 5-fold decrease in mRNA levels of *CCL5* and *IP-10* compared to mock-treated cells (Figure 7B). Furthermore, induction of *CCL5* and *IP-10* in response to pdA:dT and HT-DNA was drastically decreased in cells lacking STING or G3BP1, compared to strong induction in WT cells (Figure 7C). To determine if damage to DNA by Doxorubicin similarly activates *IFNβ* signaling and cytokine production, and the role of PERK, we first analyzed GFP-IRF3 nuclear translocation in WT, G3BP1 KO, and STING KO cells in response to Taxol and Doxorubicin. IRF3 nuclear localization was suppressed in both G3BP1 KO and STING KO, compared to WT cells, which correlate with decreased transcription of *IFNβ* and *ISG56* in KO cells compared to WT cells (Figure 7D–F). Inhibiting PERK with GSK inhibitor reduced *IFNβ* and *ISG56* mRNA accumulation, further validating our observation (Figure 7E). Induction of proinflammatory cytokines *IP-10*, *IL-6*, *CCL5*, and *IL-8* was reduced in cells when PERK activity was inhibited by GSK compared to mock-treated cells (Figure 7E). As expected, cytokine induction was also repressed in cells lacking STING or G3BP1, suggesting a unifying mechanism of regulation of innate signaling by dsDNA of exogenous or endogenous origin (Figure 7F). Collectively, our findings show that dsDNA in the cytoplasm signals via cGAS/STING pathway and activates IFNβ signaling and cytokine production through coordination of stress response by PERK and induction of SG.

## 4. Discussion

Cytosolic nucleic acids are sensed by specialized PRRs like RIG-like receptors and cGAS/STING, which trigger innate signaling and stress response pathways, leading to the production of type I IFN and inflammatory cytokines. Previous studies have demonstrated that a crosstalk between innate immune pathways and activation of the ISR causes SG formation, leading to the amplification and regulation of type I interferon (IFN) induction in the context of cytosolic dsRNA [46]. Here, we show that dsDNA, exogenous and endogenous, engages cGAS/STING signaling pathway and triggers an ISR through the phosphorylation of eIF2α by PERK, leading to SG formation in addition to the canonical IFNβ signaling. In cells with genetic deletion or pharmacological inhibition of PERK, the SG induction was abrogated and this correlated with decrease in production of IFNβ and cytokines. Pharmacological inhibition of IRE1 did not impact SG induction by DNA ligands or DNA damage agents. Activation of the integrated stress response (ISR) depended on STING, as cells with CRISPR/Cas9-mediated STING knockout formed fewer stress granules in response to DNA ligands, Doxorubicin, or Taxol, and produced lower levels of interferons and other cytokines. G3BP1 knockout cells failed to form stress granules and showed a comparable reduction in interferon and cytokine production in response to dsDNA demonstrating an intersection of stress response and DNA sensing in innate signaling. In cells treated with Doxorubicin or Taxol, we propose that self-DNA fragments may leak into the cytosol and act as endogenous ligands for the cGAS–STING pathway, contributing to the DNA damage response, as shown by increased γH2AX levels. Furthermore, our data show that activation of cGAS–STING pathway increases oxidative stress, evidenced by higher ROS levels and transcriptional upregulation of enzymes involved in redox homeostasis, such as superoxide dismutase. Uncontrolled increase in oxidative stress can further enhance DNA damage and provide a positive feedback mechanism that may eventually result in cell death. Taken together, these findings suggest that dsDNA-driven STING activation engages PERK prior to TBK1–IRF3 signaling and its downstream transcriptional signaling. STING-dependent PERK activation phosphorylates eIF2α, promoting integrated stress response signaling and stress-granule formation, in a process that requires G3BP1. Surprisingly, STING activity was attenuated in G3BP1-knockout cells pointing to a previously unrecognized role for G3BP1 in regulating STING in a reciprocal mechanism that warrants further investigation. Overall, our study identifies a STING–PERK–G3BP1 signaling axis as a key regulator of DNA sensing and the cellular response to DNA damage-induced inflammation.

We first tested different types of exogenous dsDNA ligands and cyclic dinucleotide that are STING agonists and compared SG induction with dsRNA polyI:C (signals through RLRs). SG induction followed typical kinetics of assembly and disassembly that are expected of a dynamic liquid–liquid-phase-separated (LLPS) compartment. Multiple studies demonstrated that DNA damage induces accumulation of endogenous dsDNA in the cytoplasm that signals through cGAS and STING to induce IFNβ and cytokines. We show that both Doxorubicin and Taxol induced SG formation like dsDNA ligands, suggesting a conserved signaling mechanism involving the recruitment of stress response pathways. Of the four stress kinases that can phosphorylate eIF2α and activate integrated stress response, we focused on PKR and PERK, as both have demonstrated roles in IFNβ induction during virus infections [37,56,57]. Genetic ablation of PERK in MEFs or pharmacological inhibition reduced eIF2α phosphorylation and SG formation, while PKR lacking MEFs or cells with IRE1 inhibition could activate ISR and form SGs like in WT cells. In addition to MEFs, primary NuFF cells showed similar reduction in SG when PERK was inhibited extending broader applicability of these results to multiple cell types (Figure S2). Cytosolic DNA can bind a range of sensors like cyclic GMP-AMP synthase (cGAS), interferon gamma-inducible 16 (IFI16), absent in melanoma 2 (AIM2), DEAD-Box Helicase 41 (DDX41), and Z-DNA-binding protein (ZBP1), based on localization and features of the ligand. Our data, using siRNAs to knockdown (KD) the individual receptors, show that cGAS is the primary sensor and PRR for pdA:dT and HT-DNA in HT1080 cells. Accordingly, the cGAS KD cells showed reduced activation of STING and phosphorylation of TBK1 needed to trigger downstream signaling. Overexpression of STING activates the innate signaling pathway and phosphorylates eIF2α, as in WT cells treated with STING agonists, while STING KO cells show loss of innate signaling, supporting our observation that cGAS-STING axis activates the dsDNA sensing cascade. Furthermore, cells lacking RLR signaling, RIG-I KO, show unaltered signaling like WT cells. Based on our results, as expected, we observe decrease in SG formation in cells without STING, as they had lower levels of phosphorylated eIF2α. G3BP1 was required for SG formation as no SG was detected by staining for another SG protein, TIA1. In our previous study, we showed that cytosolic dsRNA recruited G3BP1 and innate dsRNA-binding proteins into assembly of unique antiviral stress granules that are distinct from canonical SGs and serve as a platform to enhance antiviral signaling and interferon (IFN) production [46]. While the stress kinase nucleating SG and the composition and properties of SG formed by dsRNA and dsDNA may differ, PKR vs. PERK, respectively, our studies show similar pattern of signaling by both nucleic acids.

Mechanistically, how STING activates PERK at the ER is unclear; however, a recent study showed interaction between STING and PERK induced by cGAMP resulting in PERK activation and altered translation program in organ fibrosis [36]. Whether similar mechanism of PERK activation is induced in our study remains unresolved and will be addressed in future studies. PERK can regulate STING in some contexts wherein inhibiting PERK promoted mitochondrial dysfunction and accumulation of mitochondrial DNA activating cGAS-STING [58,59]. Phosphorylation of PERK inhibited STING translocation in myeloid-derived suppressor cells, resulting in immunosuppression [58]. In traumatic brain injury model, inhibiting PERK dampened STING-mediated IFN production, alleviating brain injury and cell loss [60,61]. These results suggest that STING and PERK can mutually regulate each other in some contexts which appear to be due to differences in cell types or under pathological contexts [62]. In our studies, we observe requirement of STING for PERK activation and eIF2α phosphorylation for the SG induction and cytokine production; we have not ruled out the reciprocal regulation of STING by PERK activity.

While our studies demonstrate dsDNA-induced activation of PERK in a STING-dependent manner causing SG formation requiring G3BP1, other studies show a role for G3BP1 in enhancing the DNA-binding activity of cGAS [63,64]. In that study, cells lacking G3BP1 reduced DNA binding by cGAS and subsequent cGAMP production; however, the impact on STING activation or phosphorylation of eIF2α was not explored. The cells lacking cGAS, as would be expected, were not defective in SG formation with polyI:C, a dsRNA mimic, or arsenite, which activates another stress kinase HRI and not PERK. Also, unlike U937 cells, in HT1080 cells, we observe SG formation with ISD, suggesting cell line-specific effects may also contribute to variability. In addition to regulating SG formation and thereby IFN induction, we explored if G3BP1 may regulate STING activity by affecting its trafficking. Overexpression of STING induces IFN, and we showed that cells overexpressing HA-STING formed SG; however, STING did not co-localize with G3BP1. Additionally, HA-STING was activated by phosphorylation which was significantly reduced in the absence of G3BP1 with concomitant decrease in *IFNβ*-promoter activity with or without dsDNA treatment. In resting WT cells, STING was localized with ER marker; BiP and activation by DNA ligands resulted in aggregation of STING in speckles which no longer localized with ER marker BiP. In G3BP1 KO cells, STING remains in the ER, co-localized with BiP even after DNA ligand treatment, indicating an important role of G3BP1 in STING trafficking to ERGIC compartment. Surprisingly, in HA-STING overexpressing cells, G3BP1 is still required for STING trafficking. It is likely that G3BP1 functions at more than one level by regulating (a) cGAS DNA-binding activity and (b) STING-PERK axis, which requires further exploration.

The DNA damage response functions mainly as a genome-surveillance mechanism, but recent studies show that it also plays an important role in antitumor immunity by inducing type I IFN and cytokines in response to chemotherapy agents and ionizing radiation-induced leakage of self-DNA [65,66]. The cGAS-STING pathway is a key mediator which senses the mislocalized nuclear and mitochondrial DNA that accumulates in the cytoplasm following genotoxic stress, activating signaling cascades that promote the production of type I IFNs and other inflammatory cytokines. In this study, we show that exposure of cells to topoisomerase inhibitors, chemotherapy drugs, and oxidative stress induces phosphorylation of eIF2α, which is the nucleating step in ISR activation and SG induction, and PERK is required for this effect. Release of mitochondrial DNA (mtDNA) following mitochondrial damage in senescent cells or artificially targeting mtDNA restriction digest activated ISR and PERK [67,68]. In senescent cells, STING activated IFNβ and cytokines; however, in an acute kidney injury model, mitochondrial damage triggered STING-TBK1 and NF-κB to produce cytokines but not IRF3 and type I IFN [69,70]. However, we show using a panel of STING agonists and biochemical approaches that activation of STING, induction of ISR, and subsequent SG formation are required for IFNβ and cytokine induction. Our results suggest that mechanisms governing STING activities may vary based on cell types, context, and stimuli and highlight the role of STING in TBK1-IRF3-NF-κB transcriptional pathways in regulating translational control and SG formation during DNA-induced cellular stress.

The canonical cGAS-STING pathway responds to cytosolic DNA and DNA damage by transcriptional induction of IFNβ and cytokines. However, recent studies identify a non-canonical role of STING as a regulator of ROS homeostasis by altering the transcription of genes required for oxidative stress and ROS generation [33]. In agreement, mRNA levels of *MnSOD* are reduced by inhibiting PERK in Doxorubicin-treated cells and in STING KO or G3BP1 KO, which in turn can reduce DNA damage. In other studies, cGAMP-driven DDR signaling was activated by STING-TBK1 independent of IFNβ signaling involving ATM and p53 [34]. Sustained STING activation and oxidative stress can alter ROS levels influencing susceptibility to persistent DNA damage or the efficacy of response of cells to chemotherapy agents or radiation. Cellular insults that cause ROS accumulation can cause DNA damage by oxidizing DNA bases that, in turn, can activate non-canonical STING pathways [33]. In our studies, we have not explored if the ROS production can in turn activate STING or if STING trafficking is affected by ROS levels or if both pathways can operate simultaneously in cells. In summary, our results demonstrate an interplay between STING as a regulator of redox levels and ROS homeostasis, which can act as an amplifying loop, but not explored here, as a link between innate immunity with DNA damage response pathways.

The role of SG in the context of viral infection is well-characterized while their correlation to neurodegenerative diseases and cancer is less well-understood. In our studies, we define the mechanism for SG formation in response to stress stimuli of chemotherapy drugs like Doxorubicin and Taxol. Using diverse DNA ligands introduced exogenously, we could refine the signaling mechanism of ISR and innate signaling and draw parallels with DNA damage agents. We demonstrated that DNA damage, by causing accumulation of cytosolic dsDNA, activates ER stress and PERK activity, leading to SG formation. G3BP1 is required for this effect and we made the surprising observation that G3BP1 may also in a reciprocal mechanism regulate STING signaling, which will be explored in future studies. While not explored in this study, the nature of DNA-induced SG, their protein and RNA composition, and how it compares with dsRNA-induced SG will be of broader interest. In summary, our study highlights the intersection of stress response and DNA sensing pathway in the context of innate signaling and how modulating the STING–PERK–G3BP1 axis can improve anti-tumor chemotherapy treatments.

## Figures and Tables

**Table 1:** Primer sequences for real-time RT-PCR.

Primer	Orientation	Sequence
hIFN-β	Forward	5′-GGAGGACGCCGCATTGAC-3′
	Reverse	5′-TGATAGACATTAGCCAGGAGGTTC-3′
hCCL5	Forward	5′-CCAGCAGTCGTCTTTGTCAC-3′
	Reverse	5′-CTCTGGGTTGGCACACACTT-3′
hCXCL8 (IL-8)	Forward	5′-AAGAGAGCTCTGTCTGGACC-3′
	Reverse	5′-GATATTCTCTTGGCCCTTGG-3′
hCXCL10 (IP-10)	Forward	5′-TTCCTGCAAGCCAATTTTGTC-3′
	Reverse	5′-TCTTCTCACCCTTCTTTTTCATTGT-3′
hCXCL6 (IL-6)	Forward	5′-TGTGAAAGCAGCAAAGAGGCACTG-3′
	Reverse	5′-CACCAGGCAAGTCTCCTCATTGAA-3′
hGAPDH	Forward	5′-GCAAATTCCATGGCACCGT-3′
	Reverse	5′-TCGCCCCACTTGATTTTGG-3′
hIFIT1 (ISG56)	Forward	5′-TACAGCAACCATGAGTACAA-3′
	Reverse	5′-TCAGGTGTTTCACATAGGC-3′
hMnSOD	Forward	5′-GGCTTGGTTTCAATAAGGAACGG-3′
	Reverse	5′-ATCCCCAGCAGTGGAATAAGG-3′
hNoxa	Forward	5′-ATGCCTGGGAAGAAGGCGCGC-3′
	Reverse	5′-TCAGGTTCCTGAGCAGAAGAG-3′
mIFN-β	Forward	5′-GAAAGGACGAACATTCGGAAAT-3′
	Reverse	5′-TCCGTCATCTCCATAGGGATCT-3′
mCCL5	Forward	5′-GCTGCTTTGCCTACCTCTCC-3′
	Reverse	5′-TCGAGTGACAAACACGACTGC-3′
mIFIT1 (ISG56)	Forward	5′-AGGGCTCTGCTACAAGCAACA-3′
	Reverse	5′-TGCCAATTCTTGCACATTGTC-3′
mIL-6	Forward	5′-TAGTCCTTCCTACCCCAATTTCC-3′
	Reverse	5′-TTGGTCCTTAGCCACTCCTTC-3′
mISG15	Forward	5′-GGTGTCCGTGACTAACTCCAT-3′
	Reverse	5′-TGGAAAGGGTAAGACCGTCCT-3′
mIP-10	Forward	5′-CCAAGTGCTGCCGTCATTTTC-3′
	Reverse	5′-GGCTCGCAGGGATGATTTCAA-3′

## Data Availability

The original contributions presented in this study are included in the article/Supplementary Materials. Further inquiries can be directed to the corresponding author.

## Supplementary Materials

The following supporting information can be downloaded at https://www.mdpi.com/article/10.3390/cells15020139/s1, Figure S1: WT, *PERK*^−/−^ or *PKR*^−/−^ MEFs were transfected with 2′-3′cGAMP(5 μg/mL) or polyI:C (2 μg/mL) and 6 h later immunostained with anti-G3BP1 (green) and nuclei (DAPI, blue). Cells were examined for SG (G3BP1-foci) and analyzed by confocal microscopy. Scale bars, 20 μm Representative images of cells from two biological repeats are shown; Figure S2: Immunofluorescence analysis of Human newborn foreskin fibroblasts (NuFF) cells plated on coverslips in three independent wells of 12-well plates treated with poly dA:dT with or without PERK inhibitor GSK2656157 (5 μM), SG were stained with G3BP1 antibody (green) and nuclei (DAPI, blue) and SG quantified and plotted as the percentage of SG-positive cells (mean ± SD). Data shown includes at least ≥100 cells per treatment from 3 independent wells. The data are representative of at least three independent experiments. *** *p* < 0.001. Scale bars, 20 μm; Figure S3: HT1080 cells treated with Doxorubicin (0.8 μg/mL) or Taxol (100 nM) for 24 h or 36 h and SG were immunostained with anti-G3BP1 (green) and nuclei (DAPI, blue). Cells were examined for SG (G3BP1-foci) and analyzed by confocal microscopy. Scale bars, 10 μm Representative images of cells from three biological repeats are shown; Figure S4: (A) HT1080 cells were treated with Doxorubicin (0.8 μg/mL) or Taxol (100 nM) and cell death was assessed 24, 36 or 48 h later by Trypan Blue exclusion. Results are mean ± SE from three independent experiments. (B) WT, G3BP1 KO and STING KO HT1080 cells were treated with Doxorubicin (0.8 μg/mL) or Taxol (100 nM) for 24 h or 36 h and cleavage of PARP and cleavage of Caspase 3 in cell lysates was analyzed on immunoblots and normalized to β-actin levels. The ratios of cleaved PARP/β-actin and cleaved Caspase 3/β-actin were quantified by Image J. (C) WT, G3BP1 KO and STING KO HT1080 cells were treated with Doxorubicin (0.8 μg/mL) and cell death was assessed 24 h later by Trypan Blue exclusion. Results are mean ± SE from three independent experiments. (D) WT, G3BP1 KO and STING KO HT1080 cells were treated with Doxorubicin (0.8 μg/mL) and 36 h later the mRNA levels of *Noxa* were measured by qRT-PCR and normalized to *GAPDH* mRNA levels and plotted as fold induction. Data represents mean ± SE from three independent experiments. *** *p* < 0.001.
