# Supplementary material for: Cytosolic Immunostimulatory DNA Ligands and DNA Damage Activate the Integrated Stress Response, Stress Granule Formation, and Cytokine Production
Source: Cells. 2026 Jan 13;15(2):139. doi: 10.3390/cells15020139 (PMC12839087; doi:10.3390/cells15020139)
Supplement: Supplementary file 1 [file cells-15-00139-s001.zip › cells-4053394-supplementary.pdf]

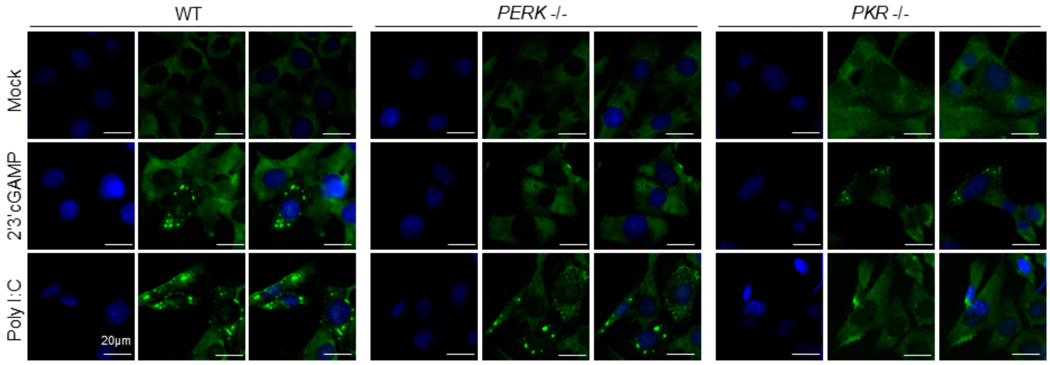

Figure S1: WT, *PERK*$^{-/-}$ or *PKR*$^{-/-}$ MEFs were transfected with 2′-3′cGAMP(5 μg/mL) or polyI:C (2 μg/mL) and 6 h later immunostained with anti-G3BP1 (green) and nuclei (DAPI, blue). Cells were examined for SG (G3BP1-foci) and analyzed by confocal microscopy. Scale bars, 20 μm Representative images of cells from two biological repeats are shown.

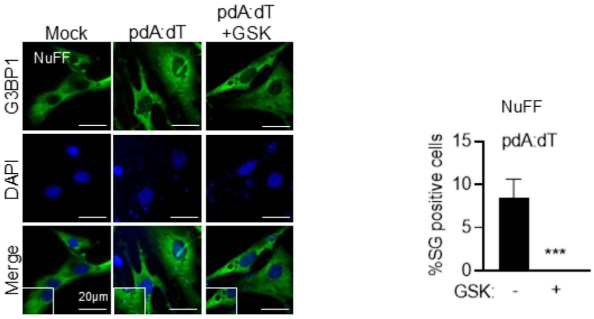

Figure S2: Immunofluorescence analysis of Human newborn foreskin fibroblasts (NuFF) cells plated on coverslips in three independent wells of 12-well plates treated with poly dA:dT with or without PERK inhibitor GSK2656157 (5 μM), SG were stained with G3BP1 antibody (green) and nuclei (DAPI, blue) and SG quantified and plotted as the percentage of SG-positive cells (mean ± SD). Data shown includes at least ≥100 cells per treatment from 3 independent wells. The data are representative of at least three independent experiments. *** $p < 0.001$. Scale bars, 20 μm.

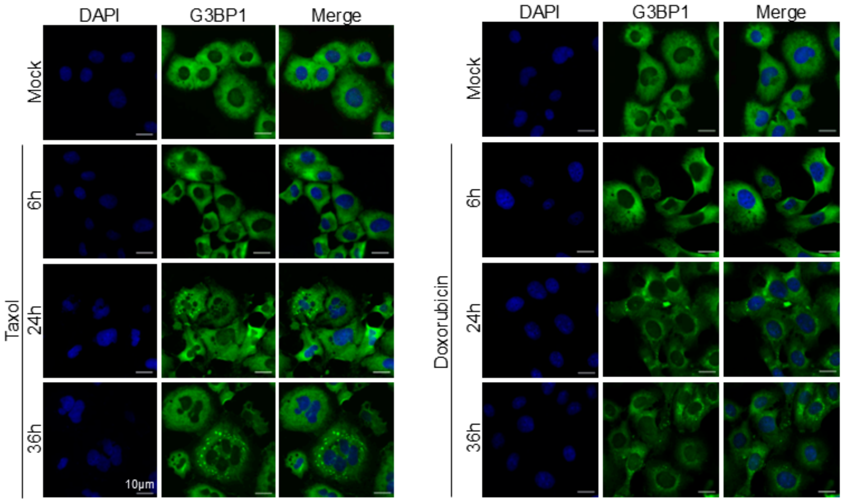

Figure S3: HT1080 cells treated with Doxorubicin (0.8 μg/mL) or Taxol (100 nM) for 24 h or 36 h and SG were immunostained with anti-G3BP1 (green) and nuclei (DAPI, blue). Cells were examined for SG (G3BP1-foci) and analyzed by confocal microscopy. Scale bars, 10 μm Representative images of cells from three biological repeats are shown.

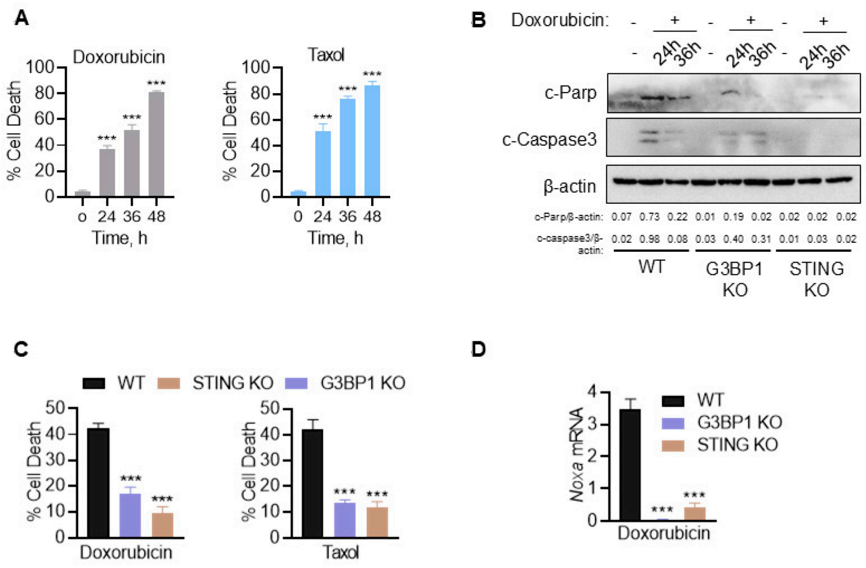

Figure S4: (A) HT1080 cells were treated with Doxorubicin (0.8 μg/mL) or Taxol (100 nM) and cell death was assessed 24, 36 or 48 h later by Trypan Blue exclusion. Results are mean ± SE from three independent experiments. (B) WT, G3BP1 KO and STING KO HT1080 cells were treated with Doxorubicin (0.8 μg/mL) or Taxol (100 nM) for 24 h or 36 h and cleavage of PARP and cleavage of Caspase 3 in cell lysates was analyzed on immunoblots and normalized to β-actin levels. The ratios of cleaved PARP/β-actin and cleaved Caspase 3/β-actin were quantified by Image J. (C) WT, G3BP1 KO and STING KO HT1080 cells were treated with Doxorubicin (0.8 μg/mL) and cell death was assessed 24 h later by Trypan Blue exclusion. Results are mean ± SE from three independent experiments. (D) WT, G3BP1 KO and STING KO HT1080 cells were treated with Doxorubicin (0.8 μg/mL) and 36 h later the mRNA levels of *Noxa* were measured by qRT-PCR and normalized to *GAPDH* mRNA levels and plotted as fold induction. Data represents mean ± SE from three independent experiments. *** $p < 0.001$.
